# The Synergy between Complex Channel-Specific FIR Filter and Spatial Filter for Single-Trial EEG Classification

**DOI:** 10.1371/journal.pone.0076923

**Published:** 2013-10-18

**Authors:** Ke Yu, Yue Wang, Kaiquan Shen, Xiaoping Li

**Affiliations:** 1 Department of Mechanical Engineering, National University of Singapore, Singapore; 2 Institute of Neurotechnology, Centre for Life Sciences, National University of Singapore, Singapore; Cuban Neuroscience Center, Cuba

## Abstract

The common spatial pattern analysis (CSP), a frequently utilized feature extraction method in brain-computer-interface applications, is believed to be time-invariant and sensitive to noises, mainly due to an inherent shortcoming of purely relying on spatial filtering. Therefore, temporal/spectral filtering which can be very effective to counteract the unfavorable influence of noises is usually used as a supplement. This work integrates the CSP spatial filters with complex channel-specific finite impulse response (FIR) filters in a natural and intuitive manner. Each hybrid spatial-FIR filter is of high-order, data-driven and is unique to its corresponding channel. They are derived by introducing multiple time delays and regularization into conventional CSP. The general framework of the method follows that of CSP but performs better, as proven in single-trial classification tasks like event-related potential detection and motor imagery.

## Introduction

The successfulness of common spatial pattern analysis (CSP) in the brain-computer interface applications such as motor imagery (MI) and event-related potential (ERP) detection has received considerable attentions [1]–[5]. Being a supervised method, CSP extracts a set of optimal spatial filters from labeled data, which maximize the separability between two distinct mental conditions. The filters obtained by CSP heavily rely on spatial projections. Therefore, it technically underrates the temporal/spectral information of electroencephalogram (EEG), which however plays an important role in feature extraction. To address such a pitfall, researchers have taken various steps to restructure CSP so that temporal/spectral filters are also exploited [6]–[8].

A noticeable attempt is the introduction of common spatio-spectral pattern (CSSP) [9], which constructs channel-specific temporal filters by applying time delay embedding. Given that the temporal filters of CSSP are rather basic, the common sparse spectral spatial pattern (CSSSP) marches forward by iteratively and simultaneously optimizing a complex temporal filter together with CSP spatial filters, under a regularization scheme [10]. It is noteworthy that in CSSSP, the obtained temporal filter will be equally applied to individual EEG channels. Unlike CSSP and CSSSP which are characterized by time delays, spectrally weighted common spatial patterns (SPEC-CSP) [11] and iterative spatio-spectral patterns learning (ISSPL) [12] introduce the linear time-invariant temporal filter and circulant temporal filter matrix, respectively. Both of them use Fourier transform so that the optimization of temporal filters can be carried out in the spectral domain. On the other hand, common spatio-temporal pattern (CSTP) [6] and bilinear common spatial pattern (BCSP) [7] show that temporal filters can be naturally obtained by modifying the objective function of CSP. Another interesting variant of CSP, namely analytic common spatial patterns (ACSP), implements Hilbert transform into CSP to extract complex-valued filters which already contain temporal information [13], [14].

In this paper, a method namely common complex-spatio-spectral pattern (CCSSP) is proposed to accommodate the benefits of both spatial and temporal filtering. It acquires a number of filters that are data-driven and tailored for each EEG channel. These filters are the mathematical synergy of spatial filters and high-order finite impulse response (FIR) filters, whose flexibility, if being well regularized, enables a better dissociation between two distinct mental conditions and consequently outstanding classification performance.

## The Proposed Method

### 2.1 FIR Filter

Suppose 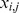 is the 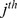 sample in the 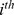 channel of multi-variant EEG matrix **X**. If a FIR filter of (2k+1) order is applied, a sample after being filtered will be
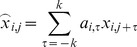
(1)where 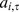 are the FIR filter coefficients that are specific to the 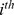 channel and 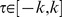. (1) can be further rewritten in the matrix format as follows:
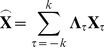
(2)where 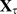 is the 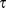 time-point delayed version of 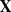, and 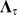 is a diagonal matrix with the 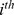 diagonal element being 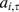.

### 2.2 Filtering

CSP aims to maximize the difference between signals of two conditions after spatial filtering. Defining 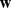 as the desired spatial filter, the objective function of CSP in this circumstance can be stated as
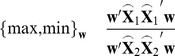
(3)where 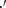 stands for the transpose operator and 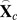 is the EEG matrix in condition 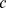 after FIR filtering. Here multiplying (2) by 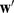 will yield
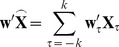
(4)where 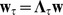. (4) can be further reorganized as
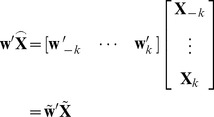
(5)Inserting (5) into the objective function (3) gives
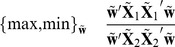
(6)


### 2.3 Singular Problem

The optimization of 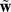 in (6) is actually equivalent to solving a generalized eigenvalue problem, just like conventional CSP [7], [15]:
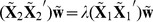
(7)However in (7), 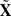 is a matrix generated by concatenating several 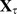. Thus it becomes practically possible that the number of ‘channels’ will overwhelm the number of samples in each channel. Such an imbalance could cause 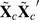 to be singular. The singularity, especially in cases where training data are very limited, can induce a biased outcome. Hence, in order to enforce a more trustworthy result, (7) shall be regularized:
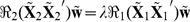
(8)where 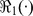 and 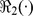 are regularization terms.

### 2.4 Regularization

It is known that conventional CSP is sensitive to both noises and overfitting [1], [16]. These disadvantages can be addressed by introducing regularization terms into CSP, which has been well discussed in [17]. In this work, an efficient and effective strategy is employed to realize regularization:
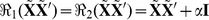
(9)where 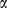 is a relatively small scalar and 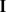 is an identity matrix. (9) will assure the uniqueness of 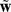.

### Experimental Setup

Two types of datasets were collected for the assessment of the proposed method. One was acquired from publically available BCI competitions datasets for motor imagery classification, and the other was obtained from self-conducted ERP detection experiments in the scenario of rapid serial visual presentation (RSVP) [6], [18], [19].

### 3.1 MI Datasets

Three publically available datasets, i.e. data set IVa, data set IIIa from BCI Competition III [20], and data set IIa from BCI competition IV (http://bbci.de/competition/iv/), recorded subjects' EEG signals while imagining their limb movements. Each dataset contained training sets and testing sets. A brief summary of the experimental paradigms pertaining to this work was presented in Table 1, whilst full details of these three datasets were available in the literature [17], [21]–[23].

**Table 1:** Brief summary of MI datasets.

Dataset	Channels	MI	Subjects	Training trials	Testing trials
IVa	118	right hand versus right foot	aa	168	112
			al	224	56
			av	84	196
			aw	56	224
			ay	28	252
IIIa	60	left hand versus right hand	k3b	45	45
			k6b	30	30
			l1b	30	30
IIa	22	left hand versus right hand	A01–A09	72	72

### 3.2 RSVP Experiments

**Figure 1:**
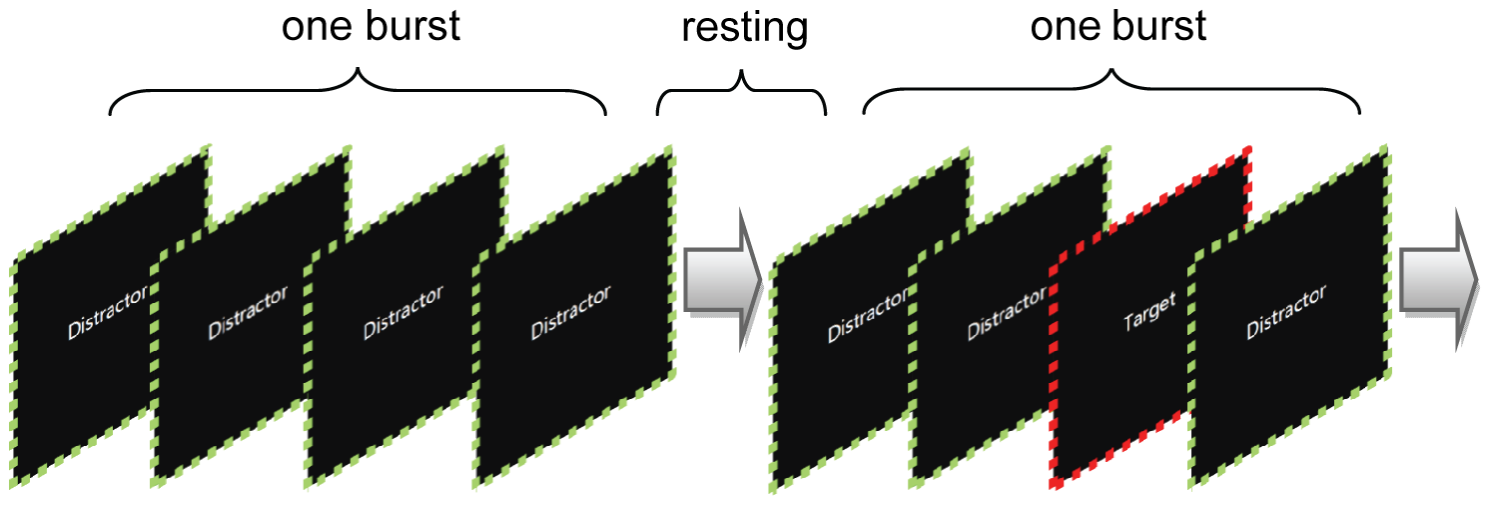
RSVP paradigm. One burst consisted of 50 images, each of which was presented for 100-sec resting period between consecutive bursts. The target image was highlighted here.

The experiments approved by the National University of Singapore Institutional Review Board (NUS-IRB) consisted of training sessions and testing sessions. In each session, a sequence of small-sized images (400×400 pixels) was presented to the subject, who was instructed to immediately press a button when images of interest (targets) appeared. In this work, the targets were images containing target objects and others were regarded as distractors. There were 41 targets and around 4000 distractors in each session. After providing their written consent forms which were approved by NUS-IRB, 4 subjects participated into the experiments. Scalp EEG signals were collected at 250 Hz, using a 62-channel ANT amplifier (ANT B.B., Enschede, Netherlands), referenced to linked ears and grounded to the forehead. Figure 1 demonstrates the experimental paradigm.

### 3.3 Preprocessing

For all three MI datasets, the preprocessing procedure followed the work in [17]. That is, each trial extracted from the time segment located from 0.5 s to 2.5 s after the cue was bandpass filtered in 8–30 Hz by a fifth-order Butterworth filter. In RSVP experiments, without analog filter, the EEG signals were firstly high-pass filtered twice and then low-pass filtered using the ‘eegfilt’ function from EEGLAB [24], with the cut-off frequencies being 1 Hz and 25 Hz, respectively. The filtered signals were segmented into an event-locked window from the onset of each image to 500 ms after the onset.

### Evaluation

Whether the proposed coupling of the high-order channel-specific temporal filter with spatial filter could render better single-trial classification capability than conventional CSP which relies solely on spatial filter, can be verified based on the overall performance on MI datasets and RSVP experiments. Additionally, results of competing methods, i.e. CSSSP, BCSP, and ACSP, were also reported for comparison.

### 4.1 Feature Extraction

Like conventional CSP, the features extracted by each method are the log-variances of the filtered signals. Only filters associated with the largest or smallest eigenvalues are used for extracting discriminative features. In the scenario of MI datasets, 3 pairs of features corresponding to the most discriminative filters were used [3], [17]. For RSVP experiments, 2 pairs of features were extracted [5], [7].

### 4.2 Classifier

The classifier adopted was weighted support vector machine (WSVM) based on LIBSVM [25] [26]. WSVM imposes higher penalties on the misclassification of the minority class [27]. Hence it is less vulnerable to the unbalanced classification problem, e.g. the number of distractors overwhelmed that of targets in RSVP experiments. For the similar reason, the balanced accuracy (BA) was particularly chosen as the performance measure for RSVP experiments in this work [6]. On the other hand, the performance measure for MI datasets resembled the work in [17].

### 4.3 Parameter Selection

As indicated in Section II, two parameters are left undetermined, i.e. order of the FIR filter 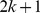 and regularization scalar 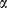. In this work, their values were chosen in two ways: 1) 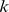 and 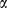 were given (5 and 10^−5^, respectively) and applied to all data sets; 2) 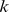 and 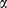 were automatically selected among [0, 1, 3, 5] and [10^−4^, 10^−5^, 10^−6^], respectively, using a 5-fold cross-validation procedure.

## Results

In Table 2, CCSSP with and without automatic parameter selection were represented by Pcv and Pfix, respectively. It can be seen that both Pcv and Pfix outperformed the conventional CSP. In specific, Pcv and Pfix achieved 2.7% and 3.7% higher average accuracy, respectively. Their performances were more superior in RSVP experiments, where the achieved accuracies were 7.9% and 8.1% higher than CSP, respectively. Among 21 subjects, Pcv and Pfix had better performance than CSP in 15 subjects. Moreover, the paired *t*-test showed that the better performance of Pcv over CSP seemed to be marginally significant (*p*-value = 0.06), and the improvement offered by Pfix was shown to be statistically significant, as *p*-value is less than 0.001. Additionally, the proposed method surpassed other methods, i.e. CSSSP, BCSP, and ACSP, according to Table 2, which was also statistically significant, with *p*-value<0.001. Moreover, the general performances of CSSSP, BCSP, and ACSP were worse than that of CSP. However, this phenomenon was absent in the scenario of RSVP experiments, where BCSP obtained 5.8% higher average accuracy in comparison to CSP, and ACSP and CSSSP had slightly better performance than CSP.

**Table 2:** Classification accuracies in % (standard deviation).

	IVa					IIIa			IIa									RSVP				
	aa	al	av	aw	ay	k3b	k6b	l1b	A01	A02	A03	A04	A05	A06	A07	A08	A09	R1	R2	R3	R4	Average
**CSP**	69.6	98.2	**67.3**	84.8	83.7	**100**	67.2	**98.3**	81.9	**58.3**	95.8	**72.9**	60.4	68.7	79.1	95.1	**93.7**	79.1	87.4	77.9	83.1	81.1(12.8)
**CSSSP**	66.9	98.2	62.2	77.2	68.6	95.5	55.1	95.0	86.1	52.0	86.1	65.9	68.0	66.6	75.0	95.1	93.0	80.8	85.4	81.3	81.3	77.9(13.7)
**BCSP**	51.8	78.5	58.6	66.8	70.7	78.8	63.7	76.6	70.8	50.0	61.8	55.5	49.3	56.2	57.6	63.1	76.3	82.2	**95.6**	83.8	89.2	68.4(13.3)
**ACSP**	65.1	96.4	65.3	79.9	76.1	76.6	56.8	51.6	90.2	52.0	95.1	69.4	56.9	70.1	78.4	**97.2**	91.6	81.4	84.6	80.2	86.1	76.3(14.3)
**Pcv**	73.2	**100**	64.7	85.7	82.9	**100**	**68.9**	96.6	**96.5**	49.3	97.2	68.0	**77.0**	**71.5**	80.5	95.8	92.3	**84.4**	91.0	91.6	91.8	83.8(13.7)
**Pfix**	**76.7**	**100**	65.3	**91.0**	**85.7**	**100**	67.2	**98.3**	93.0	56.9	**99.3**	67.3	76.3	70.1	**85.4**	95.8	92.3	83.5	91.3	**92.8**	**92.1**	**84.8**(13.0)

## Discussion

**Figure 2:**
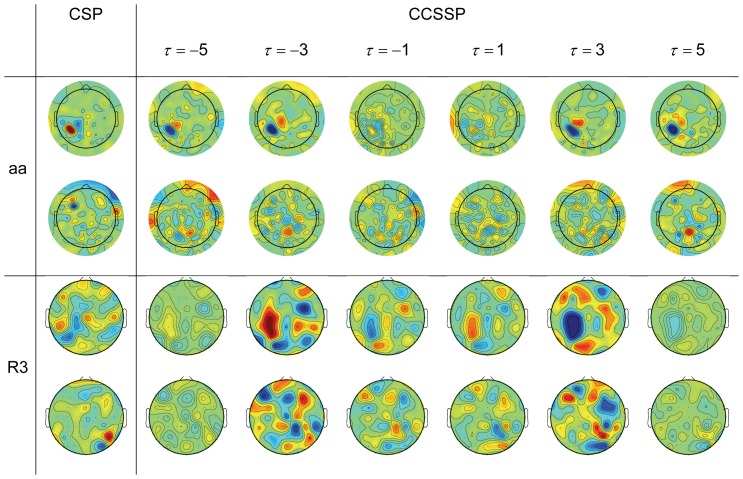
CSP filters and CCSSP spatial-FIR filters for Subject aa and R3. Spatial-FIR filters can be separated into a number of spatial filters, each of which corresponds to a 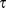 time-point delayed EEG data. The colourbar ranges from −0.5 to 0.5.

Intuitively, the difference between CSP and the proposed method lies on the types of filters that are extracted. CSP filters are purely spatial filters, whilst the proposed CCSSP exploits the more complicated, spatial-FIR filters. It is worth noting that spatial-FIR filter is essentially a combination of several spatial filters, each of which takes effect on its corresponding time-delayed EEG data. Therefore, a spatial-FIR filter can be split up into a set of spatial filters. For instance, given 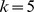 in this work, 11 spatial filters could be derived, which were partly shown in Figure 2.

For Subject aa in Figure 2, the filters at the first row, regardless of CSP or CCSSP, all imposed heavy weights on the left somatosensory area, which is in accordance to the fact that the characteristic EEG signals are controlateral to the imagined hand movement [28]. On the other hand, in the scenario of imagined right foot movement, the characteristic area localizing on the central region between left and right hemispheres [28], seemed to be overlooked by CSP filter, which however was observable in filters at 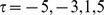. This indicates that CCSSP could uncover distinctive spatial distributions which might have been obscured by CSP. It is accomplished by exploring the temporal information of individual channel in addition to the overall spatial projection. Furthermore, Subject R3 visually presented a much prominent contrast between CSP filters and the spatial-FIR filters in Figure 2. Typically in a RSVP experiment, the main component of ERP elicited by a target, is P300, which emerges and propagates across the scalp from frontal to parietal, and has strong correlation in neighboring regions [5], [29]. Hence, it is interesting to see that the CCSSP filters contained large patches of heavy weights in parietal area as well as other regions, while the CSP filters appeared more moderate, absent from showing discriminative regions.

**Figure 3:**
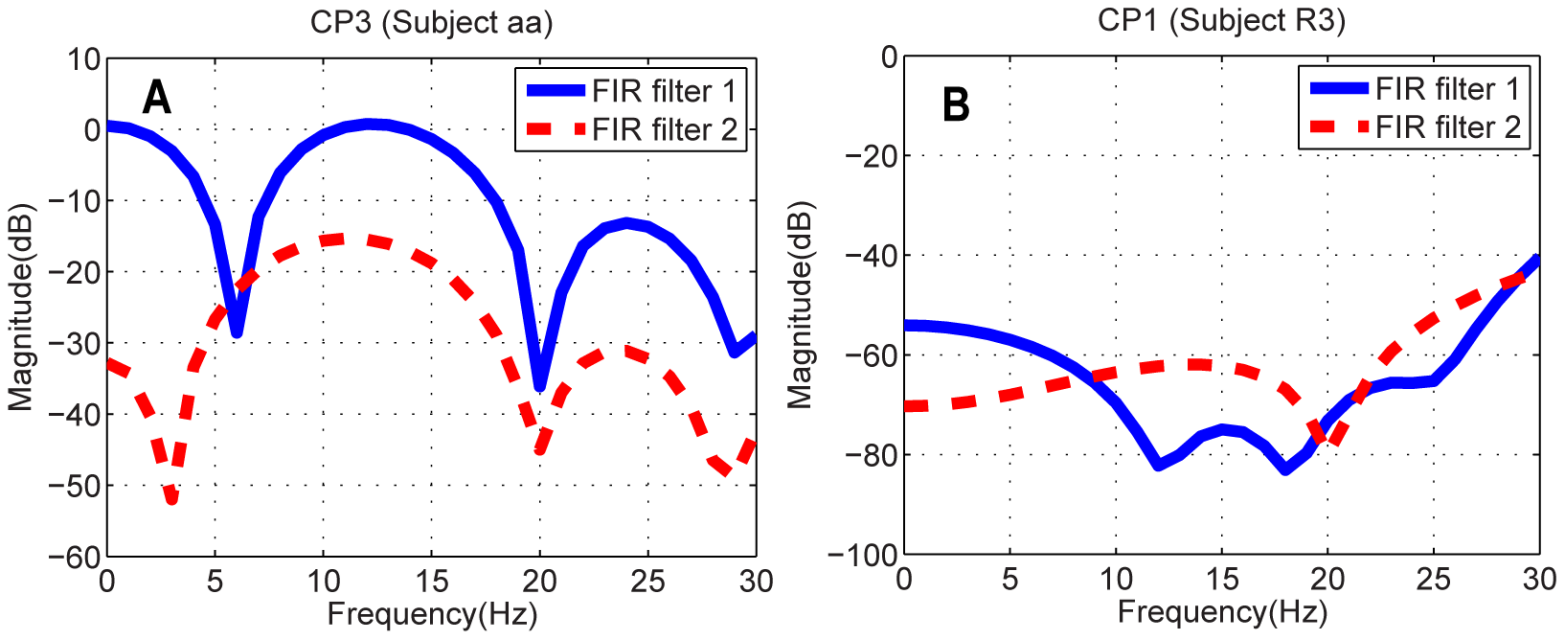
The frequency responses of FIR filters in Subject aa's CP3 and Subject R3's CP1. FIR filter 1 and FIR filter 2 correspond to the highest and the lowest eigenvalues, respectively.

Besides being perceived as a mixture of spatial filters, a spatial-FIR filter can be interpreted as a pool of channel-specific FIR filters. The fact that these FIR filters are channel-specific can be seen in Figure 2, where the changes of spatial weighting over different time delay 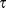 at different channels are different. It is explainable as the FIR filter coefficients defined in (1) are not uniformly, but specifically assigned to each channel. In the view of the mathematics, the selection of a channel-specific FIR filter is determined by whether this particular FIR filter can help extremize the objective function (6). Its corresponding physical meaning in this study is that, such a particular FIR filter is a filter which makes the characteristic frequency band of that channel more prominent as compared to other bands. Figure 3 shows the frequency responses of FIR filters in Channel CP3 for Subject aa and Channel CP1 for Subject R3. Specifically, the frequency response in Channel CP3 (see Figure 3A) indicated the brain oscillations at 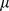 rhythm (8–12 Hz), which corresponds to the imagined movements, resides within the pass-band of FIR filter 1. Unlike FIR filter 1, FIR filter 2 relatively suppressed 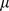 rhythm in CP3. This difference is understandable as the FIR filters were synthesized for the purpose of further differentiating two conditions, e.g. right hand v.s. right foot, where the characteristic areas are distinct from each other. It is worth noting that slow ERP of low frequency such as P300 are the signature in RSVP experiments. Although the magnitude appeared small in Figure 3B, FIR filter 1 functioned like a low-pass filter in the window of 0 Hz to 20 Hz. On the other hand, FIR filter 2 relatively suppressed low-frequency signals and emphasized more in a higher frequency band (from 10 Hz to 20 Hz).

**Figure 4:**
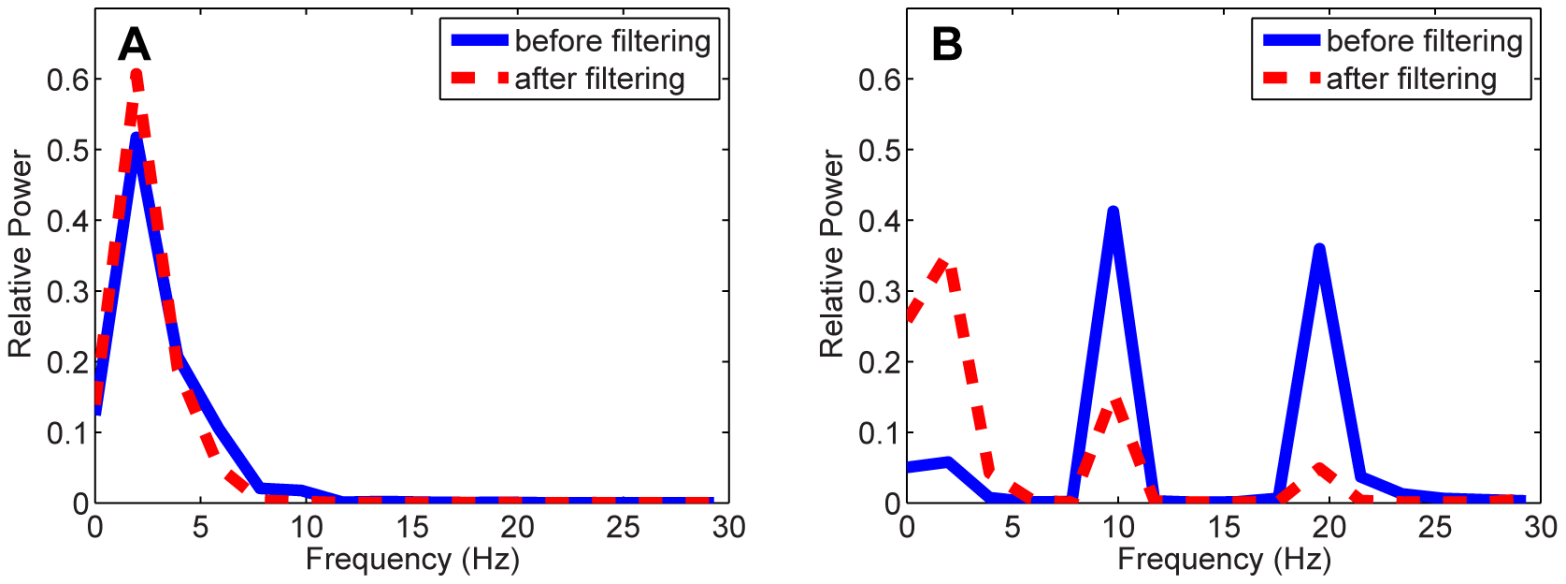
The relative power of ERP signals before and after being filtered by FIR filter 1 in **Figure 3B**. (A) shows the result of target ERP; (B) shows the result of distractor ERP.

The effect of FIR filters in Figure 3B becomes more straightforward in Figure 4, where the relative signal powers before and after filtering in CP1 are presented. As demonstrated in Figure 4, the low-frequency components both target ERP and distractor ERP were strengthened after FIR filter 1 in Figure 3B was applied, meanwhile the signal powers approximately above 5 Hz were significantly attenuated. This phenomenon also echoes that FIR filter 1 in Figure 3B served as a low-pass filter.

**Figure 5:**
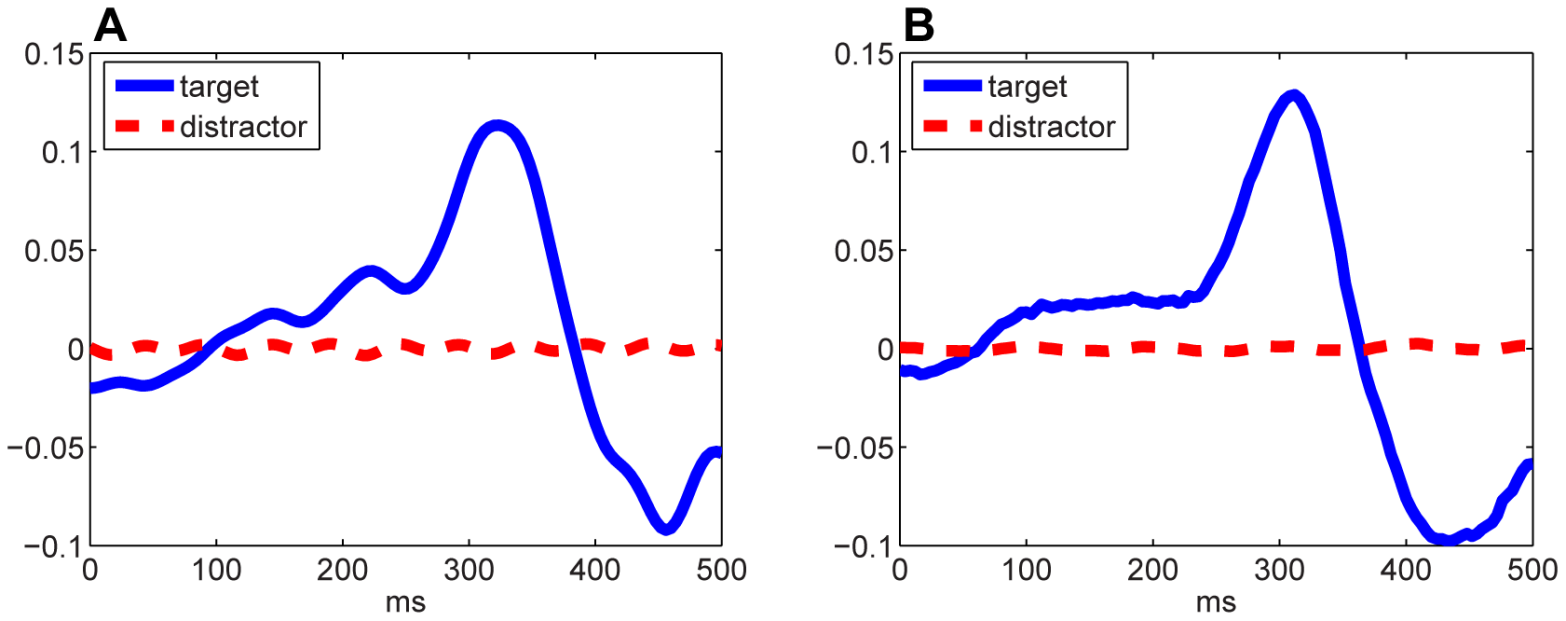
The filtered target ERP and distractor ERP. (A) shows the result of CSP filters; (B) shows the result of CCSSP filters.

Furthermore, the waveform of filtered signals could shed some light on the impact of filters on the discrimination between targets and distractors. Figure 5A and Figure 5B depict the EEG signals after being filtered by the CSP filter and the proposed spatial-FIR filter, respectively. The general waveforms in both cases were similar. That is, target ERP diverged from distractor ERP in the sense of signal power. It is noteworthy however, that the target ERP in Figure 5B had sharper and higher peak, compared to Figure 5A. In addition, the recurring ripples of distractor ERP in Figure 5A were cleaned up in Figure 5B, meanwhile the target ERP in Figure 5B suffered less up-and-downs as compared to its counterparty in Figure 5A. Such improved smoothness shall be attributed to filters like FIR filter 1 in Figure 3B which narrowed down the frequency band. Mathematically, the sharpened peak and the attenuated distractor ERP in Figure 5B indicated larger difference in variances, and consequently a better separation between two conditions.

Provided these observations and the classification performance, it could be stated that unlike CSP which tries to find stationary filters, CCSSP searches for a set of filters originated from different time delays with different weighting. This voting process could grant CCSSP the desirable flexibility and dynamics, which in return rendered better performance, in comparison to conventional CSP.

It is necessary to point out that Pcv underperformed Pfix in Table 2 in general. To a great extent, it was due to that there were insufficient training samples with regard to the number of channels of 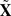 in (5). If high-density electrodes were adopted and/or accompanied by a high order of the FIR filter used, sizable training samples were required in model selection or parameter tuning. This is a noticeable drawback of the proposed method. In RSVP experiments, since there were much more training data, the selection of reliable parameters was ensured. Thus, it could be found that the performance of Pcv was comparable to that of Pfix. Another drawback of Pcv is associated with the computational burden. The matrix size of 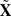 is proportional to the time delay 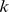. It would take remarkable time to identify the suitable regularization parameter and 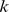.

Among other competitive methods listed in Table 2, CSSSP is the one which also makes use of the FIR filter to explore the temporal information. It optimized a single FIR filter and the single filter was applied to the entire multi-variant EEG signals, without much difference from the filters in the ordinary preprocessing step. Hence, CSSSP might improve the performance (e.g. in RSVP experiments), but the improvement could be constrained and counteracted by the necessity of careful regularization, if there was a lack of training samples (e.g. IVa, IIIa and IIa). Compared to CSSSP, BCSP performed much better in RSVP experiments, which was very close to CCSSP. However, BCSP did not perform well on MI datasets. The reason might be that, BCSP is suitable for ERP detection since ERP's time course is well defined and can be modeled in the common temporal patterns of BCSP. However, the characteristic signals of MI datasets are oscillatory rhythms, and FIR filters appeared to be more preferred. Similarly, ACSP which has evident strength in applications where phase relationships of data are critical was found not very effective on MI datasets. However, its average accuracy in RSVP experiments was slightly better than CSP. It might be partially contributed by the stronger phase relationship of ERP in comparison with that of oscillatory rhythms.

## Conclusion

In this study, CCSSP has been introduced to the CSP family. It naturally integrates and optimizes complex, specially tailored FIR filters together with spatial filters for desirable separation of two distinct conditions. The merits of such a data-driven pass-band selection for individual channels in supplemental to the broad-band CSP filtering have been attentively validated on datasets of different characteristic EEG signals. The quantitative and qualitative comparisons suggest superior discriminating capability of the proposed method over conventional CSP, e.g. 8.1% higher average accuracy in RSVP experiments.
